# Novel Agents and Emerging Strategies for Targeting the B-Cell Receptor Pathway in CLL

**DOI:** 10.4084/MJHID.2012.067

**Published:** 2012-10-09

**Authors:** Dimitar G. Efremov, Adrian Wiestner, Luca Laurenti

**Affiliations:** 1 Molecular Hematology, International Centre for Genetic Engineering & Biotechnology, Campus “A. Buzzati-Traverso”, Rome, 00016, Italy; 2 Hematology Branch, National Heart, Lung, Blood Institute, National Institutes of Health, Bethesda, MD, USA; 3 Department of Hematology, Catholic University Hospital “A. Gemelli”, Rome, 00168, Italy

## Abstract

Chronic lymphocytic leukemia (CLL) is a disease of malignant CD5+ B lymphocytes that are characterized by frequent expression of autoreactive B-cell receptors (BCRs) and marked dependence on microenvironmental signals for proliferation and survival. Among the latter, signals propagated through the BCR are believed to play a key role in leukemia initiation, maintenance and evolution. Drugs that can disrupt these signals have recently emerged as potential therapeutic agents in CLL and several of them are currently being evaluated in clinical trials. Particularly promising clinical responses have been obtained with inhibitors of the kinases SYK, BTK, and PI3Kδ, which function by blocking BCR signal transduction. In addition, recent studies focusing on the phosphatase PTPN22, which is involved in the pathogenesis of multiple autoimmune diseases and is markedly overexpressed in CLL cells, suggest that it may be possible in the future to develop strategies that will selectively reprogram BCR survival signals into signals that induce leukemic cell death. This review focuses on the biological basis behind these strategies and highlights some of the most promising BCR-targeting agents in ongoing preclinical and clinical studies.

## Introduction

The B-cell receptor (BCR) signaling pathway has recently emerged as a major therapeutic target in CLL.^1^–^3^ Signals propagated through the BCR have been shown to increase leukemic cell survival in vitro^4^–^6^ and there is growing evidence that such signals are continuously delivered to the leukemic cells in vivo.^7^,^8^ This evidence particularly refers to data obtained from gene expression profiling studies, which have shown that freshly isolated CLL cells express high levels of genes that can be induced in normal B cells by BCR engagement.^9^ Such BCR target genes are especially enriched in CLL cells isolated from lymph nodes, which is an important site of antigen encounter.^10^ In addition, the kinase SYK, which is activated immediately downstream of the BCR, is frequently phosphorylated in freshly isolated CLL cells,^11^,^12^ and this is also more prominent in CLL cells isolated from lymph nodes than peripheral blood.^10^ Lastly, surface IgM expression is downmodulated on freshly isolated CLL cells but recovers after a few days in culture, further indicating that the leukemic BCRs are continuously triggered by antigen in vivo.^13^ Altogether, these findings suggest that the BCR pathway is aberrantly or excessively activated in CLL cells and may therefore represent a promising target for therapeutic intervention.

## BCR Signals Generated in CLL Cells

**Figure 1:**
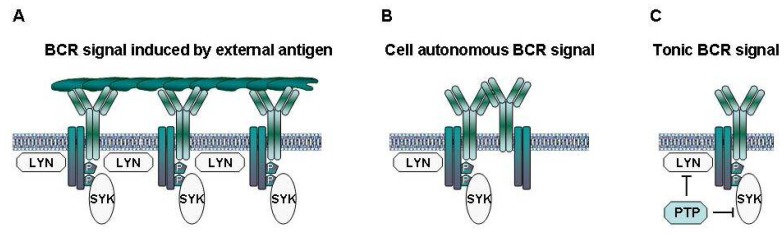
BCR signals generated in CLL and normal B cells. **A)** Binding of antigen induces aggregation of neighboring BCRs that initiate the classical antigen-dependent BCR signal. **B)** Aggregation of neighboring BCRs in CLL cells through an interaction between the CDR3 of one BCR and the FR2 of another initiates a cell autonomous BCR signal in the absence of an external antigen. **C)** Random and transient disruptions in the equilibrium between positive regulators of BCR siganling, such as the Igα-Igβ heterodimer, LYN and SYK, and negative regulators, such as the various phosphatases (PTP), could generate a tonic antigen-independent BCR signal.

Normal B lymphocytes receive two types of signals from their BCRs. The first type is triggered by binding of the BCR to external antigen, which results in the assembly and activation of a signaling complex that propagates the signal to the interior of the cell^14^ (Figure 1A). This BCR signal can induce a variety of cellular responses, including proliferation, survival, differentiation, anergy and apoptosis. The final outcome depends on several factors, such as the nature of the antigen, the availability of co-stimulatory signals and the stage of B cell differentiation.^15^ The second type of signal occurs in the absence of an external ligand and has been termed tonic BCR signal. The role of this signal in normal B cell biology has still not been fully established, but recent studies suggest that it is important for the survival of mature B cells and for normal B cell development and maturation.^16^,^17^

In recent years, evidence has emerged that both types of signals are excessively or aberrantly generated in CLL cells. For example, the kinases LYN and SYK, which are activated immediately downstream of the BCR, often shown increased basal activity in unstimulated CLL cells.^11^,^12^,^18^–^21^ This increased basal activity is maintained during prolonged in vitro culture, suggesting that it is independent of an external antigen. Increased basal activity has also been reported for other signaling molecules that are located further downstream along the BCR pathway, such as the kinases PI3K,^22^,^23^ BTK,^24^ PKC^25^ and ERK,^26^ and the transcription factors NF-kB and NF-AT.^27^–^30^ Importantly, inhibition or downregulation of many of these signaling molecules induces apoptosis in unstimulated CLL cells. Together, these findings suggest that CLL cells have an elevated tonic or basal BCR signaling activity, which contributes to their increased apoptosis resistance.

The mechanism that generates the elevated basal BCR signaling activity in CLL cells was only very recently revealed.^31^ In their seminal paper, Dühren-von Minden et al investigated a large series of CLL BCRs (n=17) and showed that all of them are capable of inducing an autonomous signal when introduced in a B cell line that does not express a functional BCR. Additional experiments demonstrated that this signal is dependent on an interaction between the heavy-chain complementarity-determining region 3 (HCDR3) of one BCR and an intrinsic motif in the framework region 2 (FR2) of another. Thus, the interaction between the HCDR3 of one BCR and the FR2 of another leads to aggregation of neighboring BCRs, which in turn generates a cell autonomous signal that is independent of an external ligand (Figure 1B).

The cell autonomous BCR signal appears to be different from the tonic BCR signal of normal B cells, as the latter occurs in the absence of any kind of ligand and is believed to be generated by transient and stochastic interactions between the BCR and the LYN and SYK kinases (Figure 1C).^16^ The cell autonomous BCR signal is also different from the extrinsic antigen-dependent BCR signal in several important aspects. For example, the cell autonomous BCR signal does not seem to result in full SYK activation, considering that phosphorylation of the activation loop tyrosines (YY525/526) of SYK is usually not detected in unstimulated CLL cells, whereas it is readily detected in most CLL samples stimulated with anti-IgM antibodies, which are used to mimic external antigen^32^. Phosphorylation of the activation loop tyrosines in SYK is required for sustained PLCγ2, AKT and ERK activation and SYK-mediated B cell proliferation.^32^ In addition, most CLL cells are in a quiescent state when isolated from the peripheral blood, but can be activated and induced to enter the G1 phase of the cell cycle by BCR engagement.^6^,^33^,^34^ Together, these data suggest that CLL cell proliferation is driven by extrinsic antigen-dependent BCR signals, whereas the role of the cell autonomous BCR signal in the pathogenesis of CLL could be to maintain the viability of the leukemic precursors until they encounter the factors that promote their expansion and subsequent malignant transformation.

The nature and identity of the antigens that drive CLL cell proliferation has still not been established, although the restricted variable region gene repertoire and the expression of stereotyped BCRs in over 30% of the cases suggest that the leukemic cells in many cases recognize similar or identical antigens.^35^,^36^ In cases with unmutated IGHV genes (U-CLL), the leukemic BCRs often bind to autoantigens that are generated or exposed on apoptotic cells.^37^–^41^ Whether or not these autoantigens drive the expansion of the malignant cells is still unclear, since U-CLL BCRs are frequently polyreactive and can also bind to microbial antigens, such as Streptococcus pneumoniae polysaccharides^37^ or the cytomegalovirus phosphoprotein pUL32.^42^ Thus, it still remains possible that the driving force for CLL cell proliferation is a chronic infectious agent, as is the case with certain indolent lymphomas that arise in the setting of chronic bacterial or viral infections, such as H. Pylori-associated gastric MALT lymphomas^43^,^44^ or hepatitis C virus (HCV)-associated splenic marginal zone and lymphoplasmacytoid lymphomas.^45^,^46^ Alternatively, the driving force could be autoantigens associated with apoptotic blebs, which co-localize with DNA- or RNA-containing complexes and can therefore provide necessary co-stimulatory signals by triggering Toll-like receptors.^47^

## Features of the BCR Pathway in CLL Cells

CLL cells have several distinct BCR signaling features that distinguish them from normal B cells. These include the previously described cell autonomous BCR signal, the low-level surface IgM expression and the heterogeneous signaling responses induced by BCR engagement in vitro. The latter are particularly evident with respect to BCR proximal signaling events, such as induction of SYK and PLCγ2 phosphorylation and intracellular Ca^2+^ mobilization, which are weak or absent in a substantial proportion of cases.^48^–^50^ These impaired signaling responses are more common in cases with favorable prognostic features, such as those that express IGHV-mutated BCRs or do not express ZAP-70.^51^,^52^ However, even in cases that appear to transduce BCR signals relatively efficiently, activation of downstream pathways can be variable and incomplete when compared to normal B cells. For example, despite efficient activation of AKT and ERK, anti-IgM induced activation of JNK, p38MAPK and NF-kB is often negligible or absent.^5^

Heterogeneous responses are also observed when the same cells are stimulated with different types of BCR crosslinking agents. For example, stimulation with soluble anti-IgM antibodies induces apoptosis in most CLL samples, whereas stimulation with the same antibodies immobilized on solid structures usually increases the survival of the leukemic cells.^5^,^6^,^53^ These data suggest that different types of antigens can induce different responses in CLL cells and indicate that the variability in the clinical course could be in part determined by the antigen specificity of the malignant B cells.

Another distinct feature of CLL cells is the aberrant expression of multiple molecules that are involved in BCR signal transduction. Most of these regulatory molecules are expressed at higher levels in CLL than normal B cells, but some show reduced expression. For example, the kinases LYN,^18^ LCK,^54^ SYK,^20^ ZAP-70^9^, BTK^24^ and PKCβ,^55^ the adaptor protein TCL1^56^ and the phosphatase PTPN22^57^ are all overexpressed in CLL cells, whereas the adaptor protein SHC^58^ and the phosphatase PHLPP1^59^ are expressed at markedly reduced levels. The functional significance of these abnormalities is still not completely clear, although at least for some of them there is evidence for a positive effect on the activity of downstream signaling pathways that regulate CLL cell survival. This particularly refers to the reduced PHLPP1 and increased TCL1 and PTPN22 expression, which result in enhanced AKT activation in anti-IgM-stimulated CLL cells.^56^,^57^,^59^ AKT is a key signaling molecule that regulates the survival of BCR-stimulated CLL cells by inducing the expression of several important antiapoptotic proteins, including MCL-1, BCL-xL and XIAP.^60^ Reduced expression of PHLPP1 could additionally increase the survival of BCR-stimulated CLL cells by allowing for enhanced activation of ERK,^59^ which regulates the activity of the proapoptotic protein BIM.^61^ Finally, overexpression of PTPN22 can have a positive effect on CLL cell survival not only by enhancing AKT activation, but also by inhibiting signaling molecules that activate proapoptotic pathways, such as p38MAPK.^57^ Together, these findings indicate that CLL cells have a very delicately fine-tuned signaling machinery that allows them to amplify weak antiapoptotic BCR signals while at the same time blocking signals that could have a negative impact on their survival. Such a machinery could be particularly advantageous to CLL cells that express autoreactive BCRs, as it could prevent their elimination by immunological tolerance mechanisms and may even allow for their positive selection by autoantigen.

## Potential Targets Along the BCR Pathway in CLL

### LYN

**Figure 2:**
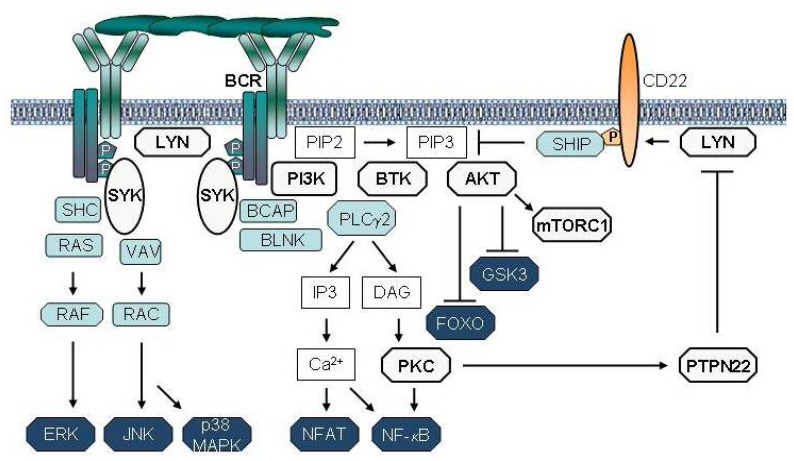
Main therapeutic targets along the BCR signaling pathway in CLL cells. Aggregation of the BCR by antigen induces the phosphorylation of the ITAMs within the cytoplasmic tails of the Ig-α/Ig-β heterodimer. Phosphorylation of the ITAMs is mediated by LYN or other members of the SRC family kinases. SYK is subsequently recruited to the phosphorylated ITAMs and activated by a multistep process that involves phosphorylation by SRC family kinases and trans-autophosphorylation. SYK further propagates the signal by phosphorylating and recruiting several signaling intermediates, such as BLNK, BCAP, VAV, SHC and PI3K. Recruitment of PI3K leads to the production of phosphatidylinositol-3,4,5-triphosphate (PIP3) and subsequent BTK and AKT activation. SYK and BTK then activate PLCγ2, which produces the second messengers inositol-1,4,5-triphosphate (IP3) and diacylglycerol (DAG). These second messengers induce the release of intracellular Ca^2+^ and activate PKC, which then activate the transcription factors NF-κB and NFAT. PKC also induces the expression of the phosphatase PTPN22, which dephosphorylates and inactivates LYN. This event prevents LYN-mediated recruitment and activation of the phosphatase SHIP, which is a key negative regulator of the PI3K/AKT pathway. In the absence of active SHIP, AKT continues to be activated by PIP3, resulting in amplification of the BCR survival signal. BCR signaling molecules that have been studied as potential therapeutic targets in CLL are depicted in grey.

Transduction of the BCR signal is a complex process that involves multiple kinases, phosphatases and adaptor proteins, which could represent potential therapeutic targets (Figure 2). The signal is initially propagated by SRC-family kinases, such as LYN, FYN or BLK, which phosphorylate the immunoreceptor tyrosine-based activation motifs (ITAMs) in the Igα and Igβ chains of the BCR. The phosphorylated ITAMs recruit the kinase SYK, which then becomes activated through SRC-family kinase-dependent phosphorylation at Y352 and trans-autophosphorylation at YY525/526. LYN not only phosphorylates and activates SYK, but it also activates phosphatases that inhibit BCR signal transduction. Thus, LYN functions as both a positive and negative regulator of BCR signaling.

As mentioned earlier, LYN is overexpressed, constitutively active and abnormally distributed in CLL cells^18^. Targeting of LYN with SRC-family kinase inhibitors, such as PP2 and Dasatinib, induces leukemic cell apoptosis.^18^,^62^,^63^ However, it is still uncertain whether the cytotoxic effect of these compounds is due to inhibition of LYN or inhibition of related kinases. For example, BTK and ABL are also inhibited by Dasatinib, and both of these molecules appear to be important for CLL cell survival.^64^–^66^ Silencing of LYN by RNA interference should clarify this issue, but so far this approach has not proven very effective presumably because of the significant overexpression and long half-life of LYN in CLL cells.^18^,^67^

### Clinical experience with LYN inhibitors

Dasatinib is an oral pan-SRC kinase and ABL inhibitor that is currently approved for the treatment of CML (Table 1). A phase 2 trial in patients with relapsed or refractory CLL treated with a daily dose of 140 mg for up to 24 months was recently published.^68^ Modest clinical activity was observed. Partial responses by NCI-WG criteria were achieved in 3 of the 15 enrolled patients (20%). Among the remaining 12 patients, 5 had nodal responses by physical exam, and one additional patient had a nodal and lymphocyte response but with severe myelosuppression. Primary toxicity was myelosuppression, with grade 3/4 neutropenia occurring in 67% of patients and grade 3/4 thrombocytopenia in 40%. Pharmacodynamic studies indicated apoptosis in peripheral blood CLL cells within 3 to 6 hours after dasatinib administration, which interestingly was associated with downregulation of SYK mRNA.

### SYK

SYK is currently one of the most attractive targets for therapeutic intervention along the BCR pathway in CLL. This kinase propagates the BCR signal by phosphorylating the adaptor proteins BLNK, BCAP and SHC, and activating key signaling intermediates, such as PI3K, BTK and PLCγ2 (Figure 2). In addition to its essential role in transducing the BCR signal, SYK is also involved in integrin, chemokine and Fc-receptor signaling.^69^

SYK is constitutively phosphorylated on the activating Y352 residue in CLL cells,^12^ presumably as a consequence of the cell autonomous BCR signal.^31^ Inhibition or silencing of SYK decreases the basal activity of downstream signaling molecules, providing additional evidence that constitutively active SYK molecules are present in CLL cells.^12^,^21^ Inhibition of SYK also induces moderate apoptosis in unstimulated CLL cells, further suggesting that the cell autonomous BCR signal increases leukemic cell survival.^12^,^19^–^21^ In addition, treatment of CLL cells with the SYK inhibitor fostamatinib completely blocks transduction of extrinsic antigen-dependent BCR signals.^12^ Fostamatinib also inhibits integrin signaling, antagonizes the protective effect of stromal cells, and reduces migration of CLL cells to chemokines and their adhesion to stromal components.^19^,^70^

Many of the in vitro effects of fostamatinib have been recapitulated in vivo using the Eμ-TCL1 transgenic mouse model of CLL.^71^ In this model, fostamatinib treatment results in inhibition of BCR signaling, an initial transient lymphocytosis, reduced proliferation and survival of the malignant B cells, and prolonged survival of the treated animals.

### Clinical experience with SYK inhibitors

Fostamatinib, also called R788, is an oral prodrug that is converted in vivo into the active compound R406.^72^ The latter is a potent inhibitor of SYK, with an IC50 in vitro of 41 nM. R406 is not entirely specific for SYK, as it also inhibits FLT3, JAK, LCK, VEGFR2 and RET at similar or slightly higher concentrations.

R406 is the first BCR signaling agent that was shown to be active in CLL.^73^ This compound was initially developed as potential treatment for allergy and rheumatoid arthritis, because of its capacity to inhibit signaling through FcɛRI and Fcγ receptors.^74^ Early trials of fostamatinib in patients with rheumatoid arthritis showed clinical activity and potent anti-inflammatory effects.^75^,^76^ Subsequently, fostamatinib was tested in a phase 1/2 trial of relapsed or refractory NHL and CLL.^77^ In the phase 1 part of the trial a dose of 200mg p.o. bid was established. Dose-limiting toxicities were neutropenia, thrombocytopenia, and diarrhea. In the Phase 2 study, 68 patients were enrolled into 3 groups according to histology, including 23 patients with diffuse large B cell lymphoma (DLBCL), 21 patient with follicular lymphoma and 24 patients with other NHL, among which 11 had SLL/CLL. The highest response rate was observed in SLL/CLL, where 6 patients (55%) achieved a partial response (PR). Objective responses were also seen in 22% of DLBCL [1 complete response (CR), 4 PRs], 10% of follicular lymphoma (2 PRs) and 11% of mantle cell lymphoma patients (1 PR). In the phase 2 portion of the trial the most common adverse events were reversible cytopenias, fatigue, diarrhea, and hypertension. All patients with CLL/SLL exhibited a transient initial lymphocytosis that has since been observed with all other agents that target the BCR pathway. This effect is believed to be caused by disruption of adhesion and retention signals delivered through the BCR, chemokine receptors and/or integrins, resulting in mobilization of CLL cells from lymph nodes and marrow into the blood.^19^,^70^

Follow-up studies with fostamatinib in CLL have not been performed to date because the company that is developing this agent is pursuing a registration strategy in rheumatoid arthritis. Clinical trials are proceeding in diffuse large B cell lymphoma, but it remains unclear whether fostamatinib will be available for future studies in CLL. However, newer more potent and more specific SYK inhibitors with promising pre-clinical activity in CLL have recently been developed and are expected to enter into clinical trials in the near future.^78^,^79^

### PI3K

PI3K is a key downstream mediator of BCR signaling.^80^ CLL cells generally express high levels of active PI3K^22^,^23^ and sustained activation of this pathway increases their proliferative capacity and survival.^60^,^81^

Several PI3K isoforms exist, some of which are ubiquitously expressed whereas others are cell type specific. PI3Kδ is the predominantly expressed isoform in leukocytes. It consists of two components, a regulatory p85 and a catalytic p110δ subunit. Upon crosslinking of the BCR, p85 binds via its SH2 domains to phospho-tyrosine motifs on CD19 and BCAP, which are a co-receptor and adaptor protein that are phosphorylated by LYN and SYK, respectively.^80^ Binding of p85 activates the catalytic p110 subunit, which phosphorylates phosphatidylinositol 4,5-bisphosphate (PIP2) to generate phosphatidylinositol 3,4,5-trisphosphate (PIP3). PIP3 then recruits the cytoplasmic kinases BTK and AKT to the cellular membrane. AKT is a key PI3K effector and following its translocation to the cellular membrane is activated by PDK1 and the mammalian target of rapamycin complex 2 (mTORC2). Active AKT then phosphorylates a number of downstream targets that are important for B-cell proliferation and survival, including the GSK3 kinase, the FOXO transcription factors and the mTORC1 inhibitor TSC2 (Figure 2). In addition to transducing signals from the BCR, PI3Kδ also plays a role in the transduction of co-stimulatory signals that originate from other receptors, such as BAFF, CD40, and Toll-like receptors.

Treatment of CLL cells with the PI3Kδ inhibitor CAL-101 (recently renamed to GS-1101) induces apoptosis in unstimulated CLL cells and inhibits survival signals generated by triggering of the BCR.^23^,^82^,^83^ In addition, CAL-101 inhibits AKT and ERK activation and abrogates the protective effects of CD40L, BAFF, fibronectin, nurse-like cells and stromal cells in vitro.^23^,^83^ CAL-101 also down-regulates secretion of chemokines and reduces CLL cell migration beneath marrow stromal cells, consistent with clinical data showing marked reductions in circulating CCL3, CCL4, and CXCL13 levels and a transient lymphocytosis during CAL-101 treatment.^83^

### Clinical experience with PI3Kδ inhibitors

CAL-101 is an orally bioavailable, potent and selective inhibitor of p110δ. In vitro, the selectivity of CAL-101 to p110δ has been reported to be IC50 of 2.5nM compared to 820, 565 and 89nM for p110α, β, and γ, respectively.^82^ In addition CAL-101 was found to be 400–4000 fold more selective against the related kinases C2β, hVPS34, DNA-PK, and mTOR, whereas no activity was observed against a panel of 402 diverse kinases at 10μM.^82^

Clinical results with CAL-101 have not yet been published, but data presented at conferences suggest promising clinical activity. At the American Society of Hematology (ASH) 2010 scientific meeting, Furman et al reported the results from a phase 1 study in 37 patients with relapsed or refractory CLL.^84^ The investigated CAL-101 dose levels were 50 mg, 100 mg, 150 mg, 200 mg and 350 mg BID, and 300 mg QD. A significant decrease in lymphadenopathy was observed, with 91% of patients having at least a 50% reduction in lymph node size. Similar to the experience with fostamatinib, an increase in lymphocytosis occurred in 60% of the patients, which was maximal during the first 2 cycles and gradually decreased thereafter. Considering traditional response criteria, partial responses were observed in 33% of the patients. Pharmacodynamic studies with peripheral blood CLL cells showed reduction of phospho-AKT to background levels after just one week of treatment.

Updated data on 54 patients were presented at the American Society of Clinical Oncology (ASCO) annual meeting in 2011. Overall response rate (ORR) by IWCLL criteria was 26%. However, 84% of the patients had a reduction in lymphadenopathy by ≥50%, but did not meet the criteria for response by IWCLL because of the increase in circulating lymphocytes^85^. Treatment was well tolerated over prolonged exposure periods exceeding one year. Grade ≥3 adverse events included pneumonia (24%), neutropenia (24%), thrombocytopenia (7%), febrile neutropenia (7%), anemia (6%), and hepatic transaminases elevation (6%). Other side effects were generally mild. Dose response assessments supported the 150 mg BID dose for future phase 2 studies.

CAL-101 is currently studied in various combinations. A recent preliminary report was presented on a phase 1 study of CAL-101 in combination with rituximab and/or bendamustine in patients with relapsed or refractory CLL^86^. All three combinations showed a favorable safety profile and more than 80% of patients receiving each regimen met IWCLL criteria for response. The lymphocyte mobilization phenomenon observed with CAL-101 monotherapy was significantly reduced, particularly with bendamustine, likely due to its rapid cytotoxic effects.

### mTOR

The mammalian target of rapamycin (mTOR) is a serine/threonine kinase with a critical role in signal transduction pathways linking growth stimuli with cell cycle progression. It is present in two complexes that share mTOR as their catalytic subunit: mTORC1, which is a downstream effector of AKT, and mTORC2, which is an activator of AKT. The defining subunits of the two mTORCs are the regulatory-associated protein of mTOR (RAPTOR) in mTORC1 and the Rapamycin-insensitive companion of mTOR (RICTOR) in mTORC2. AKT activates mTORC1 by phosphorylating the tuberous sclerosis complex 2 (TSC2), which otherwise inhibits mTORC1 activity.

In vitro treatment of CLL cells with Rapamycin (sirolimus), a highly specific inhibitor of mTORC1, inhibits cell cycle progression in CLL cells that have been induced to proliferate with CpG-oligonucleotides and interleukin-2.^87^ In addition, rapamycin prevents upregulation of survivin, a pro-survival molecule from the family of inhibitors of apoptosis proteins (IAPs) that is overexpressed in proliferative centers of B-CLL patients in vivo.^87^,^88^ Rapamycin also displayed activity in the Eμ-TCL1 transgenic mouse model of CLL, where it slowed leukemia growth and prolonged the survival of the treated animals.^89^

### Clinical experience with mTOR inhibitors

Everolimus (RAD001) is a rapamycin analog that is FDA-approved for the treatment of relapsed renal cell carcinoma. A pilot trial of everolimus in patients with advanced B-CLL demonstrated some degree of activity, but was stopped early because of toxicity concerns.^90^

A larger phase 2 study of oral single-agent everolimus (10 mg/day) was subsequently conducted in patients with recurrent/refractory indolent lymphoid malignancies, including 22 patients with CLL.^91^ Four CLL patients (18%) achieved a partial response by NCI Working Group 1996 criteria. Six additional patients (27%) achieved a nodal response with a simultaneous increase in absolute lymphocyte counts, suggesting that this agent also induces the lymphocyte mobilization phenomenon typical of BCR signaling inhibitors. Myelosuppression was more common with everolimus than with other agents that target the BCR pathway. Grade 3 or 4 anemia, neutropenia, and thrombocytopenia were reported in 23%, 32%, and 50% of patients, respectively. Five serious infections occurred, including 2 fatal, supporting the need for antimicrobial prophylaxis and growth factor support in future clinical trials with this drug.

### BTK

BTK is a cytoplasmic tyrosine kinase that belongs to the TEC kinase family. It is predominantly expressed in hematopoietic cells, particularly B lymphocytes and myeloid cells, and has an essential role in B cell development and BCR signaling. Mutations in BTK block B cell maturation at the pre B cell stage and cause X linked agammaglobulinemia (Bruton s agammaglobulinemia), an immunodeficiency disorder characterized by the virtual absence of B cells and recurrent bacterial infections.

BTK is activated upon BCR engagement in two steps. The first step is recruitment to the plasma membrane by PIP3, which is generated by PI3K. Subsequently, BTK is phosphorylated by SRC family kinases at Y551 and autophosphorylated at Y223. BTK then phosphorylates PLCγ2, which produces the second messengers inositol-1,4,5-triphosphate (IP3) and diacylglycerol (DAG). These second messengers induce the release of intracellular Ca^++^ and activate PKC, which in turn activate the transcription factors NF-kB and NFAT (Figure 2). Activation of NF-kB results in the induction of multiple antiapoptotic genes and increased cell survival.

Downregulation of BTK by RNA interference induces apoptosis in DLBCL cell lines with a chronic active BCR signal,^92^ but similar experiments have not yet been reported with primary CLL cells. However, the BTK inhibitor PCI-32765 induces modest apoptosis in primary CLL cells, which is greater than that observed in normal B cells.^24^ PCI-32765 also effectively abrogates survival signals provided to CLL cells through various microenvironmental stimuli, including BCR and integrin engagement, soluble factors (CD40L, BAFF, IL-6, IL-4, and TNF-α), and stromal cell contact.^24^ In addition, PCI-32765 inhibits proliferation of CLL cells induced by CpG oligonucleotides,^24^ presumably by blocking Toll-like receptor signals that are also transduced through BTK.^93^

PCI-32765 has similar effects on CLL cell migration and adhesion as the previously described BCR inhibitors. These include inhibition of migration towards CXCL12 and CXCL13, inhibition of CCL3 and CCL4 secretion and inhibition of α4β1-mediated adhesion to fibronectin and VCAM-1.^94^,^95^ These effects have also been recapitulated in vivo in the Eμ-TCL1 transgenic mouse model, where PCI-32765 causes a transient early increase in lymphocytosis followed by a delay in leukemia progression.^94^

### Clinical experience with BTK inhibitors

PCI-32765 (recently renamed to Ibrutinib) is a potent, orally available, and specific inhibitor of BTK, with an IC50 of 0.5 nM.^96^ It irreversibly inhibits BTK by covalently binding to its cysteine-481 residue.

The initial phase 1 study with this agent examined dose escalation in various B-cell malignancies.^97^ In this study, 47 patients were enrolled, including 15 patients with CLL. Objective responses were seen in all histological subtypes. The ORR in CLL was 60%, with 1 CR and 8 PRs. Treatment was well tolerated and drug related grade 3/4 toxicities were reported in only 9 patients (19%).

In a subsequent phase 1b/2 study, 61 patients with relapsed or refractory CLL were enrolled at 2 different doses (420 mg and 840 mg).^98^ As seen with other BCR inhibitors, a characteristic pattern of response occurred in the majority of patients, with an initial transient increase in lymphocytosis that diminished over time. The ORR by IWCLL criteria in the 420mg cohort was 70% at 10.2 months median follow-up, whereas in the 840mg cohort it was 44% but with a shorter median follow-up of 6.5 months. An additional 19% and 35% of patients in these cohorts, respectively, had a nodal PR (>50% reduction in aggregate lymph node size) with residual lymphocytosis. The ORR appeared to be independent of molecular risk features, such as high-risk cytogenetic abnormalities and unmutated IGHV genes. Disease progression occurred in only 8% (5/61) of patients during treatment; 6-month progression free survival (PFS) was 92% in the 420mg cohort and 90% in the 840mg cohort. Grade 3 adverse events considered potentially related to PCI-32765 treatment occurred in 21% of patients.

PCI-32765 is also currently being evaluated in a Phase 1b/2 trial of elderly (>65 years old) previously untreated CLL/SLL patients.^99^ Preliminary data show an ORR by IWCLL criteria of 73% (19/26), including 2 CRs, at a median follow-up of 10.7 month. Adverse effects were minimal and estimated 12 month median PFS was 93%, suggesting that PCI-32765 could be an effective and well tolerated therapeutic option for elderly patients with CLL.

Like CAL-101, PCI-32765 is currently being evaluated in combination with chemoimmunotherapy in patients with relapsed or refractory CLL. High OR rates of 90% and 100% were recently reported for the combinations PCI-32765/bendamustine/rituximab and PCI-32765/ofatumumab, respectively.^100^,^101^ Both combinations were well tolerated.

Another selective, irreversible and potent BTK inhibitor with an IC50<0.5nM, called AVL-292, has been developed more recently. Preliminary data from a phase 1b trial in CLL and B-NHL have been reported.^102^ The drug was well tolerated and displayed the typical early effects of BCR inhibitors, including the initial rise in absolute lymphocyte counts and the simultaneous reduction in lymphadenopathy.

### PTPN22

PTPN22 is a protein tyrosine phosphatase that terminates antigen-receptor signaling in T and B cells by dephosphorylating the activating tyrosine residue of SRC family kinases.^103^,^104^ It is expressed solely in cells of the immune system, including T cells, B cells and dendritic cells. A common polymorphic allele of PTPN22 that carries a substitution of tryptophan for arginine at position 620 (R620W) is a major risk factor for the development of multiple autoimmune diseases, including insulin-dependent diabetes mellitus, rheumatoid arthritis, and systemic lupus erythematosus. Interestingly, the risk allele inhibits more strongly antigen-receptor signaling than the wild-type protein. Reduced BCR and TCR signaling in autoreactive B and T cells could be a mechanism to circumvent autoantigen-induced negative selection, indicating that the risk allele contributes to the development of autoimmunity by blocking proapoptotic stimuli that are responsible for the deletion of autoreactive lymphocytes.^103^–^105^

PTPN22 is markedly overexpressed in CLL cells and may play a similar role in CLL pathogenesis as has been proposed for autoimmune diseases.^57^ In support of this possibility, experiments with primary CLL cells in vitro show that silencing of PTPN22 by RNA interference results in greater cell death following exposure to proapoptotic BCR stimuli, such as soluble anti-IgM.^57^ The capacity of PTPN22 to protect CLL cells from proapoptotic BCR stimuli is derived from its dual role in BCR signal transduction. On the one hand, PTPN22 blocks activation of downstream molecules that activate proapoptotic pathways, such as p38MAPK, whereas on the other hand it increases the activity of the AKT kinase, which provides a powerful survival signal to antigen-stimulated CLL cells. Thus, overexpression of PTPN22 results in a net increase in AKT activity despite the overall attenuation of the BCR signal. This selective uncoupling of AKT from other downstream BCR pathways is the result of inhibition of a negative regulatory circuit that involves LYN and the phosphatase SHIP, which is a major negative regulator of the PI3K/AKT pathway (Figure 2). Collectively, these findings suggest that PTPN22 could represent another promising therapeutic target in CLL, as reducing the amount or activity of this phosphatase would be expected to reprogram signals that increase leukemic cell survival into signals that induce leukemic cell death.

Inhibitors of PTPN22 are currently being developed by various research groups and pharmaceutical companies as potential therapeutic agents for the treatment of multiple autoimmune diseases.^106^ The main challenge with the development of these compounds is related to their insufficient selectivity and cell permeability, but recent progress in the field suggests that clinically useful compounds may become available for testing in CLL and PTPN22-associated autoimmune diseases in the near future.^107^,^108^

An alternative approach for targeting PTPN22 would be to reduce its expression. In vitro experiments have shown that PTPN22 can be induced by external stimuli that activate PKC, such as TPA or bone marrow stromal cells. Induction of PTPN22 by co-culture with bone marrow stromal cells could be efficiently blocked by the PKC inhibitors ruboxistaurin and sotrastaurin.^57^ The latter are two orally available drugs that are in advanced stages of clinical development for the treatment of various nonmalignant diseases, including diabetic retinopathy, psoriasis, and prevention of renal allograft rejection.^109^,^110^ Importantly, in experiments performed with primary CLL cells in vitro, both ruboxistaurin and sotrastaurin significantly enhanced the cytotoxic effect of soluble anti-IgM despite having no direct cytotoxic effect of their own.^57^ These data suggest that PKC inhibitors could be capable of selectively killing CLL cells in vivo by reducing PTPN22 expression, although it remains possible that the enhanced apoptosis induction observed with these agents involves also other mechanisms that are unrelated to PTPN22.

Clinical studies with PKC inhibitors in CLL have not yet been conducted, but phase 2 clinical trials are ongoing with sotrastaurin in DLBCL and with the dual PKC and AKT inhibitor enzastaurin in DLBCL, FL and mantle cell lymphoma. Considering the good tolerability and absence of significant toxic effects of ruboxistaurin and sotrastaurin, clinical studies with these PKC inhibitors in CLL appear warranted.

## Conclusion

The recent development of therapeutic agents that target the BCR pathway has generated considerable excitement both among researchers and clinicians dealing with CLL. This excitement is because these therapeutic agents can be given orally, they have minimal side effects, and they are producing very high response rates and durable responses. In addition, novel agents that target the BCR pathway continue to be developed and will hopefully allow even more selective killing of the malignant B cells. A challenge for the future will be to define the optimal drug combinations and identify the most appropriate therapeutic settings that will provide the greatest clinical benefit from this promising class of agents.

## Figures and Tables

**Table 1:** Agents that target the BCR pathway in CLL

Compound	Target	Status
Dasatinib	LYN, BTK	Phase 2 in CLL completed Approved for CML
Fostamatinib (R788)	SYK	Phase 2 in CLL completed Phase 3 in rheumatoid arthritis ongoing
PRT060318	SYK	preclinical
P505-15	SYK	preclinical
GS-1101 (CAL-101)	PI3Kδ	Phase 1 in CLL completed Phase 2/3 in CLL ongoing
Everolimus	mTOR	Phase 2 in CLL completed Approved for relapsed renal cell carcinoma
Ibrutinib (PCI-32765)	BTK	Phase 1 in CLL completed Phase 2/3 in CLL ongoing
AVL-292	BTK	Phase 1b in CLL ongoing
Ruboxistaurin	PKC	Phase 3 in Diabetic Retinopathy completed Phase 3 in Diabetic Neuropathy completed
Sotrastaurin	PKC	Phase 2 in renal transplant rejection completed Phase 2 in DLBCL ongoing
Enzastaurin	PKC, AKT	Phase 2 in Waldenström macroglobulinemia completed Phase 2 in DLBCL and FL ongoing
